# Dominance of Gas-Eating, Biofilm-Forming *Methylobacterium* Species in the Evaporator Cores of Automobile Air-Conditioning Systems

**DOI:** 10.1128/mSphere.00761-19

**Published:** 2020-01-15

**Authors:** Chulwoo Park, Hye Su Jung, Soyoon Park, Che Ok Jeon, Woojun Park

**Affiliations:** a Laboratory of Molecular Environmental Microbiology, Department of Environmental Science and Ecological Engineering, Korea University, Seoul, Republic of Korea; b Department of Life Science, Chung-Ang University, Seoul, Republic of Korea; c Research Development Division, Hyundai Motor Group, Gyeonggi, Republic of Korea; University of Wisconsin-Madison

**Keywords:** *Methylobacterium*, aggregation, air-conditioning systems, biofilm, genomes, volatile organic compounds

## Abstract

Microbial communities in the evaporator core (EC) of automobile air-conditioning systems have a large impact on indoor air quality, such as malodor and allergenicity. DNA-based microbial population analysis of the ECs collected from South Korea, China, the United States, India, and the United Arab Emirates revealed the extraordinary dominance of Methylobacterium species in EC biofilms. Mixed-volatile organic compound (VOC) utilization and biofilm-forming capabilities were evaluated to explain the dominance of *Methylobacterium* species in the ECs. The superior growth of all *Methylobacterium* species could be possible under mixed-VOC conditions. Interestingly, two lifestyle groups of *Methylobacterium* species could be categorized as the aggregator group, which sticks together but forms a small amount of biofilm, and the biofilm-forming group, which forms a large amount of biofilm, and their genomes along with phenotypic assays were analyzed. Pili are some of the major contributors to the aggregator lifestyle, and succinoglycan exopolysaccharide production may be responsible for the biofilm formation. However, the coexistence of these two lifestyle *Methylobacterium* groups enhanced their biofilm formation compared to that with each single culture.

**IMPORTANCE** Air-conditioning systems (ACS) are indispensable for human daily life; however, microbial community analysis in automobile ACS has yet to be comprehensively investigated. A bacterial community analysis of 24 heat exchanger fins from five countries (South Korea, China, the United States, India, and the United Arab Emirates [UAE]) revealed that *Methylobacterium* species are some of the dominant bacteria in automobile ACS. Furthermore, we suggested that the predominance of *Methylobacterium* species in automobile ACS is due to the utilization of mixed volatile organic compounds and their great ability for aggregation and biofilm formation.

## Abstract (precis)

Air-conditioning systems (ACS) are indispensable for human daily life; however, microbial community analysis in automobile ACS has yet to be comprehensively investigated. A bacterial community analysis of 24 heat exchanger fins from five countries (South Korea, China, the United States, India, and the United Arab Emirates [UAE]) revealed that *Methylobacterium* species are some of the dominant bacteria in automobile ACS. Furthermore, we suggested that the predominance of *Methylobacterium* species in automobile ACS is due to the utilization of mixed volatile organic compounds and their great ability for aggregation and biofilm formation.

## INTRODUCTION

The term “air conditioner” originally means a heating, ventilation and air-conditioning (HVAC) system for maintaining good indoor air conditions, but these days, it is generally used as a system only for cooling air and regulating humidity to make comfortable environments in the narrower sense (1). These systems are easily found anywhere, including the home, school, office, hospital, and even outside, and they have become an indispensable necessity for human daily life where the weather is hot. In particular, air-conditioning systems (ACS) are installed in almost all kinds of mobile cars, and they not only make drivers and passengers comfortable but also reduce the risk of accidents by improving the driver’s concentration (2). However, automobile ACS do not always offer only comfortable and fresh air, and drivers and passengers frequently complain that automobile ACS blow unpleasant-smelling air. It has often been reported that microbial growth in the evaporator core (EC) of automobile ACS is a major cause of the unpleasant-smelling air in the passenger cabin (3, 4). In addition, some microorganisms associated with automobile ACS may cause adverse health effects, such as lung impairment, allergic reactions, such as hypersensitivity pneumonitis, or infections to drivers and passengers (5, 6). Therefore, several studies on microbial communities in ACS have been performed using culture-based and culture-independent approaches, and these results showed that Methylobacterium and Sphingomonas spp. are frequently identified in automobile ACS (3, 5–8).

Dust particles or volatile organic compounds (VOCs) in the air may be important nutrient and carbon sources for microbial growth in automobile ACS. In addition, microbes in the air are an important source of the microbial community in automobile ACS. Because environmental conditions such as temperature, humidity, dust particles, VOCs, and the microbial population of the air are different, depending on the operation place of automobile ACS, microbial communities in automobile ACS may be different depending on the country. One study on the bacterial community of automobile ACS using culture-dependent and PCR-based single-strand conformation polymorphism approaches showed the dominance of *Rhizobiales*, including *Methylobacterium* species (3).

*Methylobacterium* species are ubiquitous bacteria found in domestic, clinical, natural, and artificial environments (9–11). In addition, *Methylobacterium* species occupy a large proportion of microbial populations in harsh environments (e.g., bathrooms, drinking water, and the phyllosphere) (12–14). The reason for this is not clear, but the excellent colonization ability of *Methylobacterium* species was suggested as one of the possibilities for their population dominance (12, 13). Bacteria produce biofilms, mainly composed of exopolysaccharides (EPS), extracellular DNA, and glycoproteins, to protect themselves from several environmental stresses under different environments. Some *Methylobacterium* species showed the highest aggregation capability among *Sphingobium*, *Xenophilus*, Rhodococcus, and *Methylobacterium* species isolated from drinking water; furthermore, *Methylobacterium* species combinations with Sphingobium, Xenophilus, and *Rhodococcus* also produced a large amount of biofilm (15). Increased biofilm formation and aggregation of Methylobacterium oryzae CBMB20 induced by a high C/N ratio showed promoted stress resistance, such as resistance to UV irradiation, desiccation, starvation, heat/cold shock, and salt stress (16).

The other representative characteristic of this methylotrophic bacteria is their versatile ability to degrade several organic pollutants. It is well known that Methylobacterium extorquens DM4 possesses the ability to degrade a toxic halogenated organic compound, dichloromethane (DCM), by the conversion of DCM to formaldehyde (17). Genes associated with the degradation of toluene, naphthalene, acrylonitrile, phenyl acetate, and nitriloacetate in the genome of Methylobacterium radiotolerans have been discovered, suggesting a potential role for versatile aromatic decomposition (18). In fact, several studies have reported that the predominant *Methylobacterium* species of the bacterial community in the phyllosphere can survive due to the metabolic abilities of various VOCs, including the C_1_ compounds methanol and chloromethane (19). The dominance of Sphingomonas and *Methylobacterium* species in shower curtains was also explained by their ability to utilize a wide range of carbon sources. Bath products and bath area volatiles are potential food resources for both species; in addition, biofilms play a protective role in this environment (20).

In the present study, we applied a barcoded 454-pyrosequencing strategy to analyze and compare microbial communities in the ECs of automobile ACS collected from South Korea, China, the United States, India, and the United Arab Emirates (UAE) and sought to elucidate the reason for the predominance of *Methylobacterium* spp. in bacterial communities derived from the ECs of automobile ACS.

## RESULTS

### Microbial diversities in the ECs of automobile ACS in different countries.

A total of 484,328 bacterial sequencing reads were generated from the pyrosequencing of 34 16S rRNA gene PCR amplicons. After removing low-quality and chimeric sequencing reads, 231,648 high-quality bacterial sequencing reads with an average of 6,756 numbers of reads per sample were obtained (Table 1). Because statistical community diversity indices, including the number of operational taxonomic units (OTUs), Shannon-Weaver index, and Chao1 index, are affected by the number of sequencing reads used for diversity index calculations, high-quality bacterial sequencing reads were normalized to the lowest numbers of 1,961 bacterial reads, and their statistical diversity indices were calculated using normalized sequencing reads. OTU, Shannon-Weaver, and Chao1 indices of bacterial sequencing reads representing bacterial community diversity in the ECs of automobile ACS were highly different depending on samples within the countries, but they were generally highest in Indian samples and next in UAE samples (see Fig. S1A in the supplemental material and Table 1). The box plot analysis of Chao1, a representative α-diversity index, for the bacterial sequencing reads clearly showed that the bacterial community in the ECs of automobile ACS operated in India was significantly more diverse than those in South Korea, China, and the United States (Fig. S1B). The box plot analysis also showed that the bacterial communities in UAE samples were a little more diverse than those in South Korea, China, and the United States, and the bacterial community diversities in the ECs of automobile ACS operated in South Korea, China, and the United States were relatively similar. However, bacterial community diversity indices were not related to car type or mileage (Table S1).

**TABLE 1:** Summary of pyrosequencing data and statistical analysis of bacterial communities in the ECs of automobile ACS^*a*^

Sample	No. of reads	No. of high-quality reads	Avg read length (bp)	No. of OTUs	Data by diversity index		
					Shannon	Chao1	Evenness
K1	53,571	22,880	486	170	3.29	330	0.64
K2	6,889	3,473	475	245	4.11	428	0.75
K3	15,504	6,753	473	239	3.92	447	0.72
K4	46,006	21,106	472	219	3.98	492	0.74
K5	10,375	5,403	486	314	3.72	547	0.65
K6	4,822	2,340	489	283	3.99	468	0.71
K7	23,410	10,252	481	184	3.4	325	0.65
K8	8,694	4,197	495	296	4.24	470	0.74
K9	12,610	7,451	480	277	4.12	512	0.73
K10	9,884	4,525	490	252	3.46	420	0.63
K11	6,051	3,632	498	218	2.99	510	0.56
K12	13,151	5,825	490	349	4.42	668	0.76
K13	4,818	2,632	485	355	3.68	835	0.63
K14	20,634	11,763	494	293	3.93	591	0.69
K15	18,248	8,793	479	355	4.61	622	0.78
K16	28,851	11,927	464	187	3.9	310	0.74
K17	43,141	21,090	479	211	3.86	395	0.72
K18	4,157	**1,961**	474	324	4.25	592	0.74
C1	16,131	8,756	478	336	4.36	698	0.75
C2	8,508	4,361	476	258	4.11	506	0.74
C3	14,649	7,660	495	318	3.79	750	0.66
C4	15,283	8,423	492	415	4.72	851	0.78
C5	13,743	7,789	496	377	4.66	710	0.79
C6	7,867	3,320	482	293	4.23	534	0.74
C7	6,872	3,177	480	222	3.47	366	0.64
A1	8,441	3,401	477	393	4.44	688	0.74
A2	5,743	2,275	484	279	3.75	491	0.67
A3	9,298	3,759	475	342	4.36	585	0.75
I1	9,042	4,586	471	508	4.67	1,107	0.75
I2	8,378	4,261	476	793	5.61	2,239	0.84
I3	6,372	3,398	471	832	5.77	2,317	0.86
U1	7,508	3,471	452	414	4.75	761	0.79
U2	9,719	4,355	475	405	4.56	737	0.76
U3	5,958	2,653	454	538	5.04	1,133	0.8

a High-quality sequencing reads were normalized to the lowest number of sequencing reads (1,961, in bold), and OTUs, Shannon-Weaver, Chao1, and evenness indices were calculated from normalized sequencing reads.

10.1128/mSphere.00761-19.1 FIG S1 Analysis of bacterial diversity and abundance in the ECs of automobile ACS operating in different countries. (A) The rarefaction analysis of bacterial 16S rRNA gene sequences was carried out using the RDPipeline with a 97% OTU cutoff value. (B) Box plots of Chao1 were constructed for the analysis of bacterial diversity using the package ggplot2 in the R program. *, *P < *0.05; **, *P < *0.01; ***, *P < *0.001. (C) LEfSe analysis of differential bacterial groups between Korean, Chinese, and U.S. and Indian and UAE samples was conducted. Significance levels were *P < *0.05, and only bacterial groups with a LDA score > 4.0 are shown. (D) Box plots shows relative abundance of *Methylobacterium* species by country between Korean, Chinese, and U.S. and Indian and UAE samples identified through the LEfSe analysis. *, *P < *0.05; **, *P < *0.01; ***, *P* < 0.001, significance of differences between countries. Download FIG S1, TIF file, 2.5 MB. Copyright © 2020 Park et al. 2020 Park et al. This content is distributed under the terms of the Creative Commons Attribution 4.0 International license.

10.1128/mSphere.00761-19.6 TABLE S1 Information of automobile cars used for heat exchanger aluminum fin sampling from the ECs of automobile ACS in different countries. Download Table S1, PDF file, 0.09 MB. Copyright © 2020 Park et al. 2020 Park et al. This content is distributed under the terms of the Creative Commons Attribution 4.0 International license.

### Dominance of *Methylobacterium* species in ACS.

**FIG 1:**
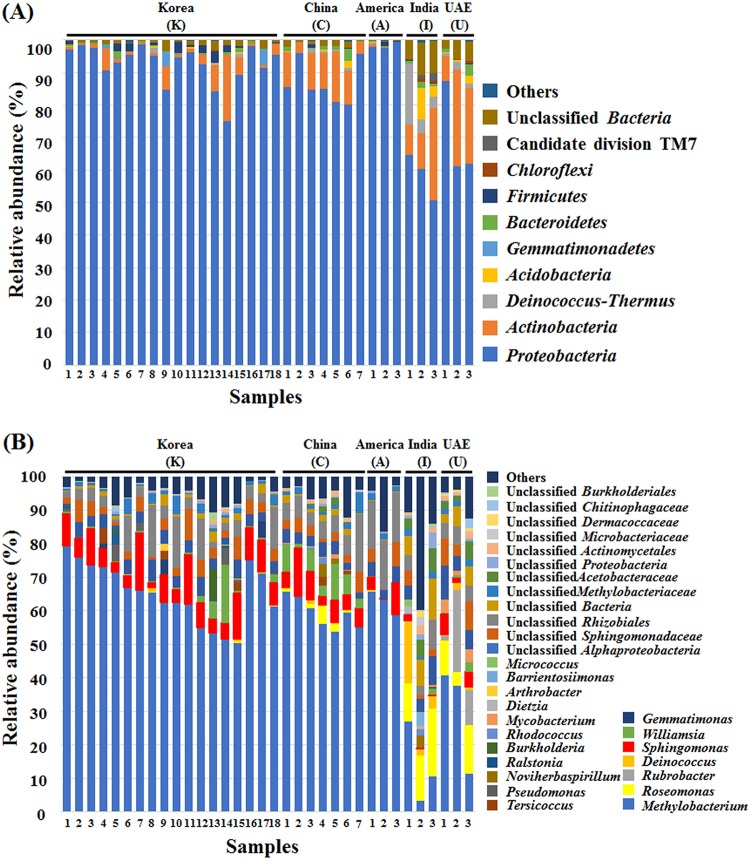
Bacterial taxonomic compositions in the ECs of automobile ACS. (A and B) Bacterial taxonomic compositions in the ECs of automobile ACS operated in different countries at the phylum (A) and genus (B) levels. Others are composed of phyla, each showing a <2.0% of the total reads in all EC samples.

The normalized bacterial sequencing reads were classified at the phylum and genus levels to investigate microbial communities in the ECs of automobile ACS operated in different countries. At the phylum-level analysis of bacterial sequencing reads, members of the phylum *Proteobacteria* were predominant, accounting for 50.1% to 99.3% of total sequences, in all EC samples regardless of country (Fig. 1A), which was in line with a previous result (21). Members of the phylum *Proteobacteria* were more abundant in Korean, Chinese, and U.S. samples, with a mean relative abundance of 91.8% ± 6.5%, than in Indian and UAE samples, with a mean relative abundance of 64.3% ± 11.2%. The phylum-level analysis of bacterial sequencing reads showed that the phyla *Actinobacteria* with 0% to 29.6% abundance and *Deinococcus*-*Thermus* with 0% to 18.8% abundance were the next dominant after *Proteobacteria*, but their abundances were quite varied depending on the samples. The phyla *Actinobacteria* and *Deinococcus*-*Thermus* were identified more abundantly from Indian and UAE samples, with mean relative abundances of 18.3% ± 9.1% and 5.2% ± 6.2%, respectively, than were those from Korean, Chinese, and U.S. samples, with mean relative abundances of 4.6% ± 5.2% and 0.2% ± 0.43%, respectively.

**FIG 2:**
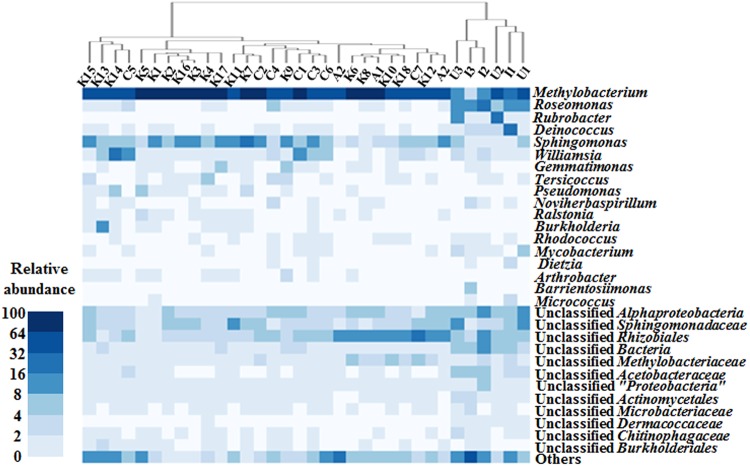
Heatmap analysis of bacterial communities in the EC of ACS. Heatmap analysis of bacterial communities in the EC of ACS operated in different countries at the genus level. Hierarchical cluster was generated based on the average linkage method with Euclidean distance measure. The color scale represents the relative abundance of each taxon on ECs.

The genus-level analysis of bacterial 16S rRNA gene sequences showed that members of the genus *Methylobacterium* (phylum *Proteobacteria*) were predominantly identified from all samples, in agreement with the results from previous studies (3, 6). However, their relative abundances were highly varied, with a range of 3.3% to 79.4% of the total bacterial sequences depending on the samples. *Methylobacterium* members were identified more highly from Korean, Chinese, and U.S. samples than in Indian and UAE samples, with mean relative abundances of 63.6% ± 7.7% versus only 21.7% ± 14.2%, respectively. The community analysis showed that *Sphingomonas* was a common genus identified from the ECs, and the relative abundance of *Sphingomonas* spp. was 6.4% ± 4.3%. The genera Roseomonas, Rubrobacter, and Deinococcus were somewhat identified from only Indian and UAE samples. However, the genera *Rubrobacter* and *Deinococcus* were barely detected from Korean, Chinese, and U.S. samples (Fig. 1B). A hierarchical clustering analysis was performed using the relative abundances of the bacterial communities (Fig. 2). The bacterial communities of Korean, Chinese, and U.S. and Indian and UAE samples fell into respective clustering groups, and the Korean, Chinese, and U.S. and Indian and UAE groups were distinctly differentiated from each other, which suggests that the air environments or operating conditions of ACS in South Korea, China, and the United States are similar to each other but different from those of India and the UAE. However, 31 out of 34 samples clearly showed that *Methylobacterium* species were the dominant bacteria in the bacterial community from ACS (Fig. 2).

A linear discriminant analysis (LDA) effective size (LEfSe) algorithm for the identification of statistically differential bacterial groups between Korean, Chinese, and U.S. and Indian and UAE samples clearly showed that the genera *Methylobacterium* and *Sphingomonas* were more abundant in Korean, Chinese, and U.S. samples than in Indian and UAE samples, whereas the genera *Roseomonas*, *Rubrobacter*, *Deinococcus*, and Barrientosiimonas were more abundant in Indian and UAE samples than in Korean, Chinese, and U.S. samples (Fig. 2 and dS1C). In particular, members of *Methylobacterium*, the most predominant bacterial genus group in the ECs, were significantly more abundant in Korea-China samples than in Indian and UAE samples, and the genera *Roseomonas* and *Rubrobacter* were significantly more abundant in Indian and UAE samples than in Korea-China samples (Fig. 2 and S1C and D).

### Superior growth of *Methylobacterium* species from the EC of ACS in mixed VOCs.

Atmospheric VOC profiles of four large cities in the Republic of Korea demonstrated that toluene is the highest concentration of VOC in the air from all four cities and ethyl acetate is the second most serious air pollutant in Daegu and Seoul (Table 2). In addition, washer liquid used in automobiles contains a high concentration of methanol (range, 30% to 90%) (22). Thus, the growth of bacteria derived from automobile ACS in the above-mentioned three VOCs was assessed. A total of 91 species of the 628 species isolated from car evaporators were selected in the following phyla: *Alphaproteobacteria* (34 species), *Betaproteobacteria* (10 species), *Gammaproteobacteria* (1 species), *Bacteroidetes* (5 species), *Firmicutes* (8 species), and *Actinobacteria* (33 species), without duplicated species (Table S2). Among them, 12 species of *Methylobacterium* were identified. Gordonia species showed the highest growth under all three single VOCs at each time point (Fig. S2A). However, the growth of *Methylobacterium* species was not surprisingly increased in toluene-supplemented minimal salt basal (MSB) medium, except for Methylobacterium aquaticum (Fig. S2A). Five of the 12 species of *Methylobacterium* (M. aquaticum, M. rhodesianum, M. brachiatum, M. radiotolerans, and M. isbiliense) showed higher optical density at 600 nm (OD_600_) values than the average at 144 h, but the remaining species (M. frigidaeris, M. currus, M. organophilum, M. longum, M. platani, M. dankookense, and M. aerolatum) exhibited OD_600_ values the same as or lower than the average (Fig. S2A). Unlike toluene, many automobile ACS-derived bacteria were able to utilize ethyl acetate (average OD_600_ at 144 h, toluene, 0.24; ethyl acetate, 0.33; methanol, 0.23). Among them, three *Methylobacterium* species (*M. rhodesianum*, *M. brachiatum*, and *M*. *aquaticum*) showed higher-than-average growth of ACS-isolated bacteria, but the OD_600_ values of the other five *Methylobacterium* species (*M. dankookense*, *M. aerolatum*, *M. currus*, M. platani, and *M. longum*) were below 0.2 (Fig. S2A). Although *Methylobacterium* species are well known as C_1_ compound (e.g., methanol and methylamine) utilizers, *M. currus*, *M. radiotolerans*, *M. frigidaeris*, *M. dankookense*, *M. brachiatum*, *M. isbiliense*, and *M. aerolatum* did not grow sufficiently even until 144 h. Only *M. rhodesianum* and *M. aquaticum* showed remarkable growth in OD_600_ compared to that of the other bacteria at 144 h (Fig. S2A). In addition, these species started to grow at 96 h, which is relatively slow compared to the growth under toluene and ethyl acetate.

**TABLE 2:** Main VOCs contained in the air of four Korean cities

Major VOC	Concn (ppb) in:			
	Daegu	Busan	Seoul	Incheon
Toluene	10	1.3	2.2	0.6
Benzene	0.3	0.3	0.2	0.3
Ethylbenzene	0.3	0.5	0.4	0.1
*o*-Xylene	0.3	0.6	0.1	0.1
Ethyl acetate	0.5	0.2	0.6	—^*a*^

a —, not detected.

10.1128/mSphere.00761-19.2 FIG S2 Measurement of bacterial growth isolated from ACS in VOC-supplemented medium. (A) Optical density at 600 nm (OD_600_) was measured when automobile ACS-derived bacteria were cultured under 0.5% toluene-, 0.5% ethyl acetate-, and 0.5% methanol-supplemented conditions at 144 h. Pink dots indicate the OD_600_ value of *Methylobacterium* species. (B) Growth of *Methylobacterium* species in single- or mixed-VOC-supplemented medium was measured. The OD_600_ values of *Methylobacterium* species were compared at 24, 48, 72, and 96 h when single or mixed VOCs (0.5% mixture [0.17% toluene, 0.17% ethyl acetate, 0.17% methanol] and 1.5% mixture [0.5% toluene, 0.5% ethyl acetate, 0.5% methanol]) were added. Dark-blue, orange, gray, yellow, light-blue, and green bars represent *M. currus*, *M. radiotolerans*, *M. frigidaeris*, *M. organophilum*, *M. dankookense*, and *M. brachiatum*, respectively. Download FIG S2, TIF file, 1.2 MB. Copyright © 2020 Park et al. 2020 Park et al. This content is distributed under the terms of the Creative Commons Attribution 4.0 International license.

10.1128/mSphere.00761-19.7 TABLE S2 List of bacteria isolated from car air-conditioning evaporator systems in this study. Color indicates bacterial phylum (red, *Alphaproteobacteria*; orange, *Betaproteobacteria*; yellow, *Gammaproteobacteria*; green, *Bacteroidetes*; light blue, *Firmicutes*; dark blue, *Actinobacteria*), whereas the numbers in parentheses represent the number of bacteria belonging to the phylum. *Methylobacterium* species are highlighted in bold. Download Table S2, PDF file, 0.2 MB. Copyright © 2020 Park et al. 2020 Park et al. This content is distributed under the terms of the Creative Commons Attribution 4.0 International license.

**FIG 3:**
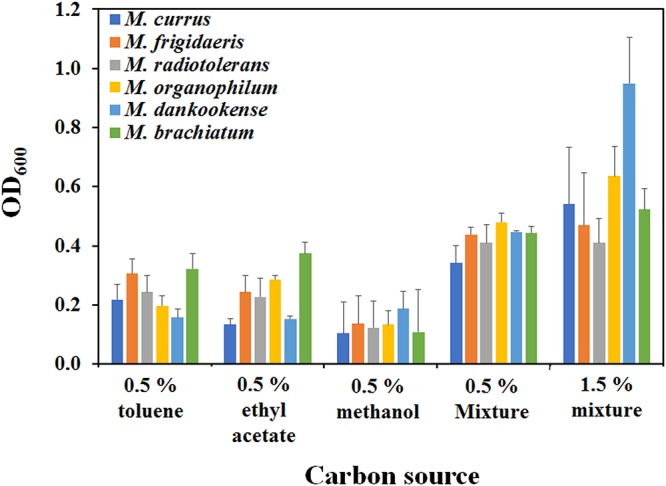
Measurement of growth under VOC-supplemented conditions. OD_600_ values of *Methylobacterium* species were compared when single VOCs (0.5% toluene, 0.5% ethyl acetate, or 0.5% methanol) or mixed VOCs (0.5% mixture [0.17% toluene, 0.17% ethyl acetate, 0.17% methanol] or 1.5% mixture [0.5% toluene, 0.5% ethyl acetate, 0.5% methanol]) were added at 144 h. Light-blue, orange, gray, yellow, dark-blue, and green bars represent *M. currus*, *M. radiotolerans*, *M. frigidaeris*, *M. organophilum*, *M. dankookense*, and *M. brachiatum*, respectively.

Because complex VOCs are present in the real world, a mixture of three VOCs was provided to *Methylobacterium* species (*M. currus*, *M. radiotolerans*, *M. frigidaeris*, *M. dankookense*, *M. brachiatum*, and *M. organophilum*) which grew poorly under a single-VOC-supplied medium. Surprisingly, enhanced growth was observed in all tested *Methylobacterium* species under the mixed three-VOC-added conditions compared to the growth when a single-carbon source was added (Fig. 3A and S2B). The most dramatically increased growth was approximately eight times at 144 h in *M. dankookense* under mixed conditions compared to that in a single-carbon source-added medium (0.5% mixture [0.17% toluene, 0.17% ethyl acetate, 0.17% methanol] and 1.5% mixture [0.5% toluene, 0.5% ethyl acetate, 0.5% methanol]) (Fig. 3 and S2B). It is notable that mixed VOCs do not have toxicity to but rather promote the growth of *Methylobacterium* species.

### Biofilm former versus aggregator lifestyles of *Methylobacterium* groups.

**FIG 4:**
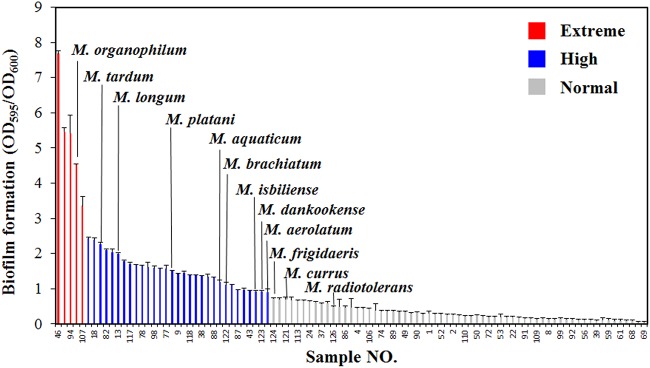
Measurement of biofilm formation among automobile ACS-isolated bacteria. Biofilm formation of 91 bacterial species isolated from automobile ACS was measured. Red, blue, and gray bars indicate extreme (OD_595_/OD_600_, >3), high (OD_595_/OD_600_, 0.9 to 3), and normal (OD_595_/OD_600_, <0.9) biofilm formers, respectively. *Methylobacterium* species are highlighted above each bar.

The growth test of 91 species on R2A medium revealed that most bacteria reached stationary phase within 60 h. In addition, the optical density values of *Methylobacterium* species at 60 h were close to or lower than the mean OD_600_ value of all tested bacteria (Fig. S4A). To compare dramatic biofilm formation, we performed a biofilm assay of automobile ACS-derived bacteria grown at 60 h in R2A medium (Fig. 4). As a result, a wide range of biofilm formation values (OD_595_/OD_600_ ratio) among automobile-isolated bacteria was observed from 7.7 to 0.07. To evaluate whether the classification of biofilm formers was valid, they were grouped as extreme (OD_595_/OD_600,_ >3), high (OD_595_/OD_600_, 0.9 to 3), and normal (OD_595_/OD_600_, <0.9) biofilm formers (Fig. 4 and S3B and Table S3). Statistically valid classifications (DF = 2, *F* = 347.8, *P < *0.001) showed that 9 of 12 *Methylobacterium* species belong to the extreme or high biofilm formers (Fig. 4 and Table S3). It was intriguing that dominance in the bacterial population is associated with the biofilm formation ability. Thus, we retrieved the community profiles, and a correlation analysis between biofilm formation and bacterial population size was conducted. The result revealed that high biofilm formers occupied a large population under automobile ACS niches (Spearman correlation coefficient, 0.23; *P < *0.05; Fig. S3C). However, there was no statistically significant difference in biofilm formation between the two groups, *Methylobacterium* and non-*Methylobacterium* species (DF = 1, *F *= 1.54, *P = *0.11; Fig. S3D). Taken together, the dominance in bacterial community is associated with the ability for biofilm formation, but this does not explain the prevalence of *Methylobacterium* species under automobile ACS.

10.1128/mSphere.00761-19.3 FIG S3 Growth of bacteria isolated from automobile ACS on R2A-rich medium at 60 h and statistical analysis of biofilm formation. (A) Represented bacterial taxonomy names indicate the highest OD_600_ value in each time. OD_600_ values of *Methylobacterium* species are marked by pink dots. OD_600_ measurements were performed in triplicate. (B) Ability of biofilm formation compared among extreme, high, and normal biofilm formers. (C) Correlation curve between population size and biofilm formation. (D) Statistical analysis of biofilm formation between *Methylobacterium* and non-*Methylobacterium* species. Download FIG S3, TIF file, 2.3 MB. Copyright © 2020 Park et al. 2020 Park et al. This content is distributed under the terms of the Creative Commons Attribution 4.0 International license.

10.1128/mSphere.00761-19.4 FIG S4 Phenotypic observation and aggregation assays. (A) Phenotypical observation of aggregators (top) and nonaggregators (bottom) of *Methylobacterium* species at 48 h on TYG medium. (B) Quantification of aggregation for *M. currus*, *M. radiotolerans, M. frigidaeris*, *M. dankookense*, *M. organophilum*, and *M. brachiatum*. Each color bar represents aggregators or nonaggregators (red, aggregators; blue, nonaggregators). Numbers above the graph represent measurement time. Aggregation percentages were calculated as described in Materials and Methods. Download FIG S4, TIF file, 1.4 MB. Copyright © 2020 Park et al. 2020 Park et al. This content is distributed under the terms of the Creative Commons Attribution 4.0 International license.

10.1128/mSphere.00761-19.8 TABLE S3 Extreme and high biofilm-forming bacteria (extreme, OD_595_/OD_600_, >3; high, OD_595_/OD_600_, 0.9 to 3). Download Table S3, PDF file, 0.1 MB. Copyright © 2020 Park et al. 2020 Park et al. This content is distributed under the terms of the Creative Commons Attribution 4.0 International license.

Although it is generally believed that aggregation is one of the essential steps toward biofilm formation, biofilm formation requires attachment to a surface, resulting in the production of a slimy extracellular matrix (15, 23). However, we observed that certain *Methylobacterium* species produced a small amount of biofilm but strongly aggregated with each other rather than attached to the surface (Fig. 4  and S4A). Like *M. currus*, aggregate formations of *M. radiotolerans* and *M. frigidaeris* were observed during their growth. Conversely, Methylobacterium dankookense and *M. organophilum* were freely distributed in the medium, like *M. brachiatum* (Fig. S4A), which belonged to the high biofilm formers (Fig. 4). To quantify aggregation, aggregation percentages were measured during the growth of the above-mentioned six *Methylobacterium* species on tryptone-glucose-yeast extract (TYG) medium (Fig. S4B). As a result, *M. frigidaeris* is the highest autoaggregator, showing 75.4%, 90.0%, and 86.8% aggregation in 12, 24, and 48 h, respectively. *M. currus* was also aggregated, with high percentages of 29.1%, 53.6%, and 50.8% at the same time intervals, respectively. Thus, *M. currus*, *M. radiotolerans*, and *M. frigidaeris* were grouped as aggregators but those that form a small amount of biofilm. *M. brachiatum*, *M. dankookense*, and *M. organophilum* were designated biofilm formers, which are relatively high-biofilm-forming species rather than aggregators (Fig. 4).

### Main factors of aggregation and biofilm formation in *Methylobacterium* species.

For a deeper understanding of aggregation and biofilm formation, comparative genome analysis was performed with genomes of *M. currus*, *M. radiotolerans*, *M. frigidaeris*, *M. brachiatum*, and *M. organophilum*. Aggregation often occurs between cells conjugated by type IV pili to exchange genomic substances (24). The numbers of genes encoding pili were varied between *Methylobacterium* species. Nine pilus-encoding genes of the *trb* operon (*trbBCDEJLFGI*) were present in the second contig of *M. currus* (Table 3). Furthermore, there were 8 genes (e.g., *tra* and *trb* genes) for the synthesis of pili in *M. radiotolerans* and 11 genes (e.g., *pilZ* and *cpa* genes) in *M. frigidaeris*. In contrast, only four genes (*trwC*, *traC*, *traG*, and *trbI*) are located in the major contig of *M. brachiatum*, and few genes could be observed in the genome of *M. organophilum*. Field emission scanning electron microscopy (FE-SEM) analysis revealed that *M. currus* was conjugated by pili that could be also observed in other aggregators (*M. radiotolerans* and *M. frigidaeris*) (Fig. S5A). However, this pilus network was not detected in the biofilm former group, including in *M. brachiatum*, *M. organophilum*, and *M. dankookense*.

**TABLE 3:** Pilus synthesis-related genes in the genomes of *M. currus*, *M. radiotolerans*, and *M. frigidaeris*

Genome	Locus tag	Annotation	Gene^*a*^
*M. currus*	MP1016A_06858	Conjugal transfer protein	*trbI*
	MP1016A_06859	Conjugal transfer protein	*trbG*
	MP1016A_06860	Hypothetical protein	*trbF*
	MP1016A_06861	Protein kinase shaggy	*trbL*
	MP1016A_06863	Hypothetical protein	*trbJ*
	MP1016A_06864	Probable conjugal transfer protein	*trbE*
	MP1016A_06865	Hypothetical protein	*trbD*
	MP1016A_06866	Conjugal transfer protein	*trbC*
	MP1016A_06867	Probable conjugal transfer protein	*trbB*
*M. radiotolerans*	MRAD2831_RS62055	Conjugal transfer protein	*traG*
	MRAD2831_RS62060	Conjugal transfer protein	*traD*
	MRAD2831_RS63110	VirB4 family type IV secretion/conjugal transfer ATPase	*—*
	MRAD2831_RS63125	Conjugal transfer protein	*traH*
	MRAD2831_RS63135	P-type conjugative transfer protein VirB9	*—*
	MRAD2831_RS63150	Conjugal transfer protein	*traG*
	MRAD2831_RS63175	Conjugal transfer protein	*trbM*
	MRAD2831_RS63225	Conjugal transfer protein	*—*
*M. frigidaeris*	CS379_RS00995	Pilus assembly protein	*—*
	CS379_RS01005	Pilus assembly protein	*—*
	CS379_RS05350	Pilus assembly protein	*pilZ*
	CS379_RS09770	Pilus assembly protein	*cpaB*
	CS379_RS11780	Pilus assembly protein	*pilZ*
	CS379_RS13205	Pilus assembly protein	*cpaD*
	CS379_RS23685	Pilus assembly protein	*—*
	CS379_RS23690	Pilus assembly protein	*—*
	CS379_RS23695	Pilus assembly protein	*—*
	CS379_RS31900	Pilus assembly protein	*tadE*
	CS379_RS31905	Pilus assembly protein	*tadE*

a —, not identified.

10.1128/mSphere.00761-19.5 FIG S5 Comparison of genotypes/phenotypes between aggregator and biofilm former groups, and schematic overview of toluene, ethyl acetate, and methanol metabolism in *Methylobacterium* species. (A) SEM analysis of both groups was conducted, and *M. currus*, *M. radiotolerans*, and *M. frigidaeris* were observed by SEM. Yellow arrows indicate pili. Magnification, ×10,000. (B) Schematic of a flagellar synthesis operon in the genomes of *M*. *currus*, *M. frigidaeris*, *M*. *radiotolerans*, *M. organophilum*, and *M. brachiatum*. (C) Motility tests of *Methylobacterium* species on 0.1% TYG agar medium. Red, aggregators; blue, biofilm formers. (D) Schematic of a succinoglycan synthesis operon in the genome of *M*. *radiotolerans*, *M. organophilum*, and *M. brachiatum.* (E) Metabolic pathways of three VOCs were predicted based on genome analysis and previous literature. The genes participating in the metabolism are represented in gray. Download FIG S5, TIF file, 16.4 MB. Copyright © 2020 Park et al. 2020 Park et al. This content is distributed under the terms of the Creative Commons Attribution 4.0 International license.

Previous research studies have suggested that motility is important for aggregation, because nonmotile cells tend to form aggregates and hold each other (25). A large gene cluster (34,183 bp) related to the synthesis of flagella is present in the genome of *M. brachiatum*; in addition, a similar gene structure also exists in the genomes of *M. organophilum* and *M. radiotolerans*. In contrast, three flagellar synthesis-related gene clusters are separated in the genomes of *M. currus* and *M. frigidaeris* (Fig. S5B). In addition, nonaggregators (*M. brachiatum*, *M. organophilum*, and *M. dankookense*) showed excellent motility, which was apparently observed at 9 days (Fig. S5C). In contrast, aggregators (*M. currus*, *M. radiotolerans*, and *M. frigidaeris*) did not move into the medium until 9 days.

Genes participating in succinoglycan synthesis (*exoA*, *exoW*, *exoB*, *exoF*, *exoP*, *exoL*, *exoU*, *exoY*, *exoO*, and *exoM*) are present in the genome of *M. brachiatum*. Succinoglycan synthesis-related genes are closely located in the chromosomes of *M. brachiatum*, *M. radiotolerans*, and *M. organophilum* but not of *M. frigidaeris* and *M. currus* (Fig. S5D). Monosaccharide profiles using high-performance anion-exchange chromatography (HPAEC) showed that high concentrations of mannose and glucose were detected from the EPS of both species (for mannose, 73.2% in *M. currus* and 63.2% in *M. brachiatum*, and for glucose, 25.3% in *M. currus* and 20.4% in *M. brachiatum*). In addition, a low concentration of fucose (0.6%) was produced from both bacteria. However, only *M. brachiatum* EPS contained 15.7% galactose (Table S4). Likewise, *Methylobacterium* species synthesize similar or different EPS among species.

10.1128/mSphere.00761-19.9 TABLE S4 Monosaccharide proportion of EPS from *M. currus* and *M. brachiatum.* Download Table S4, PDF file, 0.04 MB. Copyright © 2020 Park et al. 2020 Park et al. This content is distributed under the terms of the Creative Commons Attribution 4.0 International license.

### Enhanced biofilm formation during coculture of aggregators and biofilm formers.

**FIG 5:**
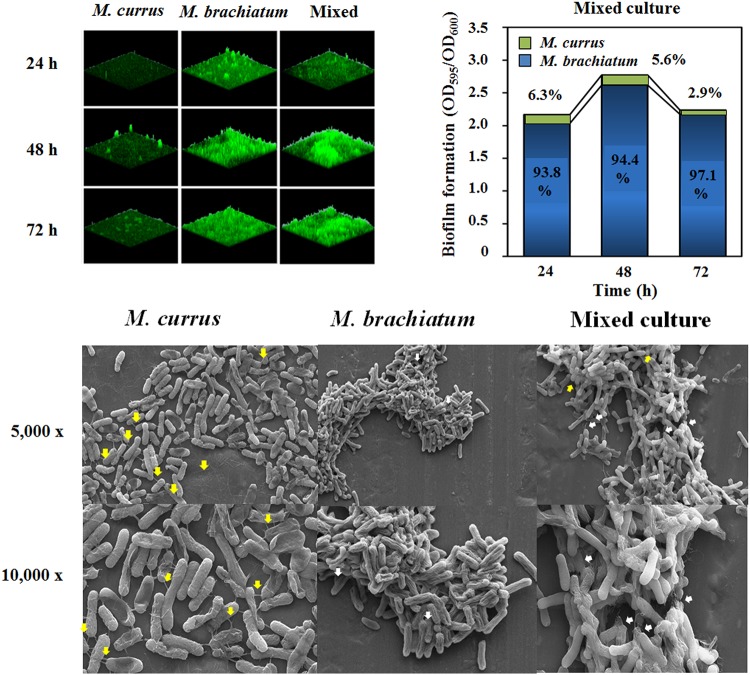
Microscopic analysis and biomass measurement of *M*. *currus* and/or *M*. *brachiatum*. (A) Biofilm formation was observed using FilmTracer Sypro Ruby biofilm matrix stain dye at 24, 48, and 72 h (left), and biofilms were quantified by a biofilm assay in mixed culture (right). The proportion of each biomass for *M*. *currus* (green) and *M*. *brachiatum* (blue) is marked on the bars when they were cultured together. (B) SEM analysis of *M. currus* and/or *M. brachiatum* and mixed culture. Yellow and white arrows indicate pili and EPS, respectively. Magnification, ×5,000 and ×10,000.

Confocal laser scanning microscopy (CLSM) analyses of *M. currus* and *M. brachiatum* during single culture or coculture showed that the biofilm production was enhanced when they were grown together rather than singly at 48 and 72 h (Fig. 5A). The investigation of the biofilm formation and cell-forming units between *M. currus* and *M. brachiatum* during coculture suggested that a low abundance of *M. currus* was maintained until 48 h (6.25% at 24 h and 5.56% at 48 h; Fig. 5A). At 96 h, *M. currus* still remained, although the amount of biofilm in mixed culture of *M. currus* and *M. brachiatum* was reduced, implying that the biofilm produced by *M. brachiatum* enables *M. currus* aggregates to attach to the surface. Furthermore, EPS from *M. brachiatum* and pili from *M. currus* could be also observed when the two species were cocultured (Fig. 5B). Taken together, biofilm formation was enhanced due to the abilities of aggregation and biofilm formation in mixed culture.

## DISCUSSION

Water vapor condensation by air cooling on ECs causes microbial growth by providing water, an indispensable life-giving factor (4). In addition, airflow through tiny air conduits of automobile ACS to efficiently cool down the air temperature leads to the deposition of microorganisms and airborne particles on the EC surface. Therefore, microbial growth, as well as the deposition of airborne microorganisms, may be an important factor in determining microbial communities in the ECs of automobile ACS. Temperature, humidity, dust particles, and VOCs that have a strong influence on the microbial growth in the ECs and microbial community in the air are different depending on the country. Therefore, in this study, microbial communities in automobile ACS that were operated in seven places in five countries were investigated using a barcoded 454-pyrosequencing strategy (26).

Microbial diversity analysis showed that bacterial communities in the ECs of automobile ACS operated in India and the UAE, especially in India, were more diverse than those in South Korea, China, and the United States (Fig. 1 and S1), which might be caused by the differences in environmental and climate factors such as temperature, humidity, and VOCs (3, 4). Because the average temperatures for the year in India (Delhi) and the UAE (Dubai) are higher than those in South Korea (Namyangju and Ulsan), China (Beijing and Shanghai), and the United States (Irvine, CA), the operation times of automobile ACS in India and the UAE will also be longer than those in South Korea, China, and the United States. The long operation time of automobile ACS will cause thick biofilm formation in the ECs with diverse microenvironments, probably giving rise to more diverse bacterial communities, which may be one of the most important for malodor production from automobile ACS (3). The EC of automobile ACS has unique environmental conditions, experiencing drastic fluctuations in temperature and water availability. It encounters high temperature by engine operation with no air-conditioner operation, whereas its temperature drops to almost zero during air-conditioner operation. When the air conditioner is not in operation, water, a necessity for microbial growth, is not provided. Dust particles or VOCs in the air may be a sole nutrient or carbon source for microbial growth in automobile ACS. Therefore, microorganisms that are able to grow in the ECs of automobile ACS may be tolerant to several stresses and have the ability to metabolize dust particles or VOCs.

The VOC profiles in the air of four Korean cities suggested that benzene-toluene-ethylbenzene-xylene and ethyl acetate were abundantly present, which is compatible with the previous literature (27). In addition, bacterial community analysis revealed that *Methylobacterium* species were one of the dominant bacteria in all ECs of automobile ACS used in this study (Fig. 1 and 2). Members of the genus *Methylobacterium* are typically strictly aerobic methylotrophic bacteria and grow well on single-carbon compounds as the sole sources of carbon and energy, as well as on a wide range of multicarbon growth substrates (28). Recently, novel *Methylobacterium* species have been isolated from ACS (29, 30), and 12 species of *Methylobacterium* were identified in the ECs of automobile ACS in this study, implying that *Methylobacterium* members might have the ability to metabolize VOCs as the nutrient or carbon source to grow in the ECs of automobile ACS.

Acetylesterase (EC 3.1.1.6) is the key enzyme that mediates ethyl acetate to acetate and ethanol in the classical ethyl acetate metabolism (31). The enzyme has been mainly discovered and studied in Pseudomonas species, so that acetylesterase (NCBI RefSeq accession no. AAM16269) in Pseudomonas putida as a query sequence was used to search the homologs with the reference genomes of *M. currus*, *M. frigidaeris*, *M. radiotolerans*, *M. organophilum*, and *M. brachiatum*. A BLASTP search showed that all *Methylobacterium* species possess the α/β-hydrolase that has more than 30% similarity and 70% query cover with the query sequence (NCBI RefSeq accession numbers WP_099953624.1, WP_099900826.1, WP_116656825.1, WP_053621636.1, and WP_091857735.1), implying that ethyl acetate metabolism could occur via acetylesterase in *Methylobacterium* species. Among five toluene-degrading pathways that have been revealed in Pseudomonas putida F1, P. putida PaW15, Burkholderia cepacia, Ralstonia pickettii PKO1, and Pseudomonas mendocina KR1 (32), *Methylobacterium* species possibly oxidize toluene to acetyl-coenzyme A (acetyl-CoA), because homologs of XylA (xylene monooxygenase electron transfer), XylB (benzyl alcohol dehydrogenase), and XylC (benzaldehyde dehydrogenase) were present in all *Methylobacterium* species (Fig. S5E). Furthermore, all five *Methylobacterium* species possess multiple copies of methanol dehydrogenases. In the case of *M. frigidaeris*, there are two copies of PQQ-dependent dehydrogenase encoding gene in the genome, which was the lowest copy number among *Methylobacterium* species. Thus, all above-mentioned homolog searches indicated that *Methylobacterium* species is a potential VOC utilizer.

However, contrary to expectations, many *Methylobacterium* spp., such as *M. dankookense*, *M. frigidaeris*, *M. organophilum*, *M. longum*, and *M. currus*, did not grow sufficiently in single-VOC-supplied media, except for two *Methylobacterium* species (*M. aquaticum* and *M. rhodesianum*; Fig. S2A). *Methylobacterium* species are well-known slow growers even in rich medium (9), suggesting that their metabolisms are slow. However, the growth of all tested *Methylobacterium* species was increased when a three-VOC mixture (toluene, ethyl acetate, and methanol) was supplied (Fig. 3). Even when the total concentrations of single and mixed VOCs (0.5%) were adjusted, the growth of *Methylobacterium* species was enhanced in the three-VOC mixture, implying that growth improvement was not due to the amount of carbon availability under VOC-mixture-added conditions (Fig. 3 and S2B). Based on VOC metabolic pathways of *Methylobacterium* species, acetyl-CoA enters the ethyl malonyl cycle after it is generated through toluene, ethyl acetate, and methanol metabolism (Fig. S5D). Thus, the activation of several genes, including those of the ethyl malonyl-CoA pathway and the tricarboxylic acid cycle, might happen during toluene and ethyl acetate metabolism, supported by the superior growth of *Methylobacterium* spp. in the sole toluene or ethyl acetate rather than in methanol-supplemented medium (Fig. 3). This activation might accelerate the growth of *Methylobacterium* species in the coexistence of VOCs (33, 34). Because it is obvious that a variety of VOCs are present in the air, improved growth under mixed-VOC-added conditions can be a reasonable explanation for the predominance of *Methylobacterium* species in automobile ACS as well as in other natural or artificial environments.

As suggested above, ACS is a harsh environmental condition due to the existence of several stresses, and one possible feature required for growth in ECs is that bacteria have the ability to form biofilms by producing EPS (6). Although the growth of *Methylobacterium* species is not above average among bacteria, even in R2A-rich medium, the profiles of the biofilm assay showed that 9 of 12 *Methylobacterium* species belong to the extreme- or high-biofilm-former groups. However, the comparison of biofilm data between *Methylobacterium* and non-*Methylobacterium* spp. proved to be statistically invalid due to the large gap between extreme biofilm and normal biofilm formers in *Methylobacterium* species (Fig. 4 and S3D). Nevertheless, the combination of bacterial population data and the biofilm assay profile revealed that high biofilm formers predominantly occupy the microbial population in the ECs of automobile ACS (Fig. S3B).

Interestingly, all three aggregators possessed many pilus synthesis-associated genes, whereas *M. brachiatum* and *M. organophilum* did not. In addition, SEM analysis showed that *M. currus* cells were connected by pili, and other aggregators also displayed conjugation between cells, indicating that pili seem to be the main factor for aggregation (Fig. S5A). Additionally, genome analysis of *M. currus* revealed that flagellin-encoding genes were divided into three large clusters, and the genes also occupy a similar location in the genome of *M. frigidaeris* (Fig. S5B). These distant three clusters possibly delay the assembly of flagella, resulting in the lack of motility (Fig. S5B). *M. radiotolerans* showed two genotypical features of aggregators and biofilm formers. A succinoglycan synthesis operon and one large gene cluster in a flagellar synthesis operon were located in the genome of *M. radiotolerans* (Fig. S5B and D). However, pilus synthesis-related genes were present in *M. radiotolerans*. In addition, this species showed an aggregator phenotype (nonmotile and conjugation) (Table 3 and Fig. S4 and S5A and C); thus, it seems that pilus synthesis function is more important in the lifestyle of *M. radiotolerans*. Coculture of the aggregator *M. currus* and the biofilm former *M. brachiatum*, a low biofilm former, increased biofilm formation compared to that with the single culture, and EPS could also be observed (Fig. 5A and B). It is expected that *M. brachiatum* may provide the surface for *M. currus* to aggregate and, as a result, produce increased biofilm, although a low abundance of *M. currus* remains in the biofilm, which is the other reason for the dominance of *Methylobacterium* species in automobile ACS.

In this study, the microbial populations in EC samples as part of automobile ACS from five different countries (South Korea, China, the United States, India, and the UAE) were analyzed by performing high-throughput pyrosequencing. The next-generation sequencing data analyses and taxonomic classification data clearly showed the microbial community of the EC surface, and *Methylobacterium* species are commonly dominant. This result is expected due to the utilization of mixed VOCs and the great ability for biofilm formation. However, aggregator *Methylobacterium* species, such as *M. currus*, tend to form a small amount of biofilm; in contrast, biofilm formers, such as *M. brachiatum*, did not show aggregation. Comparative genome analysis showed that flagella, pili, and large membrane proteins are correlated with the aggregation of *Methylobacterium* species. Finally, when aggregators and nonaggregators are cultured together, biofilm production was promoted. These data support why *Methylobacterium* species are predominant in automobile ACS; furthermore, the data are applicable for the removal of malodor in a car so that a pleasant vehicle indoor environment can be created.

## MATERIALS AND METHODS

### Sampling of heat exchanger aluminum fins from automobile ACS in different countries.

Thirty-four heat exchanger aluminum fin samples were collected from the ECs of automobile ACS in seven different regions of five countries, namely, South Korea (Namyang and Ulsan), China (Beijing and Shanghai), the United States (Irvine, CA), India (Delhi), and the UAE (Dubai; Table 1). Table S1 presents the detailed information of the mobile cars, including the sampling country, autodismantling city (sampling city), mileage, and car type. Automobile ACS that were in operation were dismantled at Hyundai Motor service centers in each country. Small portions of aluminum fins were sampled from 10 different areas of the EC of each automobile ACS using sterilized long-nose pliers, and the sampled aluminum fins were combined. The aluminum fin samples were transported to the laboratory in South Korea in dry-ice packages.

### Genomic DNA extraction and PCR amplification for pyrosequencing.

Total genomic DNA was extracted from the aluminum fin samples using the FastDNA Spin kit for soil (MP Biomedicals, USA), according to the manufacturer’s instructions. Cell lysis was achieved by bead beating 5 g of sample two times for 30 s at a 30-s interval and speed of 6.0 m/s in a DNA FastPrep24 instrument (MP Biomedicals). The primer set BacF (5′-adaptor B-AC-9F-3′)/BacR (5′-adaptor A-X-AC-541R-3′), where X denotes unique (7 to 11) barcoded sequences, was used for the amplification of bacterial 16S rRNA (V1 to V3 variable region) genes (Table S5) (35). All PCR amplifications were performed in a C1000 thermal cycler (Bio-Rad) in a 50-μl volume containing a *Taq* polymerase mixture (Solgent, South Korea), with PCR conditions as described previously (36). The PCR products were purified using a PCR purification kit (Solgent), and their concentrations were measured with an enzyme-linked immunosorbent assay reader equipped with a Take3 multivolume plate (SynergyMx; BioTek, USA). The composite sample for the community analysis of bacteria was prepared by pooling equal amounts of the PCR products and sequenced using a 454 GS-FLX titanium system (Roche, Germany) at ChunLab (Seoul, South Korea).

10.1128/mSphere.00761-19.10 TABLE S5 Adaptor and barcode sequences in PCR primer sets used in this study. Download Table S5, PDF file, 0.1 MB. Copyright © 2020 Park et al. 2020 Park et al. This content is distributed under the terms of the Creative Commons Attribution 4.0 International license.

### Sequencing data processing and analysis.

Sequencing reads were sorted into respective EC samples based on their barcode sequences, and then the barcodes were trimmed using the RDPipeline tools available at the Ribosomal Database Project (RDP; http://pyro.cme.msu.edu/) (37). Low-quality sequencing reads with ambiguous base calls (“N”), with average quality scores below 25 (error rate, 0.005), or that were shorter than 300 nucleotides were also removed using the RDPipeline. Potential chimeric sequencing reads were also removed using the UCHIME ChimeraSlayer available at USEARCH of the RDPipeline (38). To compare bacterial diversity among samples, sequencing reads were normalized to the smallest read numbers by random sequencing read resampling using the sub.sample command of the mothur program (39). Normalized sequencing reads were aligned using the fast secondary-structure-aware INFERNAL aligner (40). Diversity indices, including OTUs, Shannon-Weaver, Chao1, and evenness indices, were calculated from the normalized sequencing reads at 97% similarity level using the RDPipeline. Chao1 indices of bacterial communities in countries were compared by box plot analyses using the package “ggplot2” of the R program, and the Wilcoxon signed-rank test was performed to evaluate their statistical significance (41).

Normalized bacterial sequencing reads were also taxonomically classified into hierarchical bacterial taxa at the phylum and genus levels using the RDP Naive Bayesian rRNA Classifier at an 80% confidence threshold (42). The relative abundances of bacterial taxon communities at the genus level in each EC sample were visualized using the heatmap.2 command of the R program (gplots, version 3.3.2), and their hierarchical clusterings were determined using the dist and hclust commands of the R program, based on average Euclidean distance.

### Genome sequencing and comparative genome analysis.

As described previously, the genomic DNA of the newly isolated *M. brachiatum* was extracted using the Wizard DNA purification kit (Promega) and sequenced with a PacBio 20K sequencer at ChunLab (30). *De novo* assembly of quality-filtered sequencing reads was conducted with PacBio SMRT Analysis 2.3.0. For a deep understanding of phenotypical differences, comparative genomics of *Methylobacterium* species was performed. Previously, genome analysis of *M. currus* was performed (29); however, in this study, the genome of *M*. *brachiatum* was examined. The genome sequences for five strains of the other *Methylobacterium* species (*M. currus* [accession numbers CP028843 to CP028847] *M. frigidaeris* IER25-16 [accession no. NZ_PELK00000000], *M. organophilum* DSM760 [accession no. QEKZ00000000], and *M. radiotolerans* JCM 2831 [accession no. CP001001]) were retrieved from the National Center for Biotechnology Information database.

### Growth test on VOCs.

Growth assays of automobile-isolated strains were measured at 60 and 144 h on R2A and MSB media added with 0.5% toluene, ethyl acetic acid, and methanol. Seed cultures on R2A medium were collected, and phosphate-buffered saline (PBS) washing was performed. Resuspended bacterial cells (OD_600_, 0.04) were inoculated on MSB medium added with 0.5% toluene, ethyl acetic acid, methanol, and VOC mixtures into 96-well polyvinyl chloride (PVC) microtiter plates with shaking (220 rpm). The absorbance (OD_600_) was measured using a Spark multimode microplate reader (Tecan Trading AG, Switzerland), and the average and standard deviation values were calculated from triplicates.

### Biofilm formation ability test.

Three-day cultures of car-isolated bacteria were washed with PBS and inoculated at an OD_600_ of 0.04 into fresh R2A medium in 96-well microtiter plates (BD Biosciences, USA). The plates were incubated at room temperature without agitation. After incubation, the microtiter plates were rinsed with sterile water, and then 0.1% (wt/vol) crystal violet (CV) solution was added to stain the attached cells. After staining, the CV was removed, and the wells were rinsed with sterile water. The dye was dissolved in 95% ethanol, and the absorbance of the solubilized dye at 595 nm was then determined (43).

### Aggregation assays of *Methylobacterium* species.

The measurement of aggregation was performed for *Methylobacterium* species grown in TYG medium at 48 h. At each time point at 12, 24, and 48 h, 1 ml bacterial cells was transferred to a 1.7-ml EP tube and harvested by preprophase band (PPB) centrifugation at 800 rpm for 1 min. Then, the supernatant was measured immediately by spectrophotometry (Eppendorf, Germany). The aggregation percentage was determined using the equation

$$\left(\text{1}-A_{t}/A_{0}\right)\times \text{1}00$$

where *A_t_* represents the absorbance at different time points (*t* = 24, 48, and 72 h) and *A*_0_ represents the absorbance before the centrifugation.

### EPS purification and HPAEC analysis.

EPS purification was conducted using the ethanol precipitation method. *M. currus* and *M. brachiatum* were incubated in 1 liter TYG medium for 48 h and centrifuged to obtain a clear supernatant. The volume of the ethanol supernatant was added three times and incubated at 4°C overnight. Then, the sample was centrifuged, and the pellets were washed three times with an 80% ethanol solution, followed by three washes with PBS to remove residues and then freeze drying for 3 days. The composition of freeze-dried EPS samples was analyzed via HPAEC (ICS-5000; Dionex Co., USA). The CarboPac PA-1 column (Dionex) was used with 18 mM NaOH as the solvent at a flow rate of 1.0 ml/min at 25°C. HPAEC was performed by Biosystems, South Korea.

### FE-SEM and CLSM analysis.

For observation of phenotypical morphology, *M. currus* and *M. brachiatum* cells were incubated in TYG medium for 48 h (for single culture, OD_600_, 0.04; for mixed culture, OD_600_, 0.02 for each strain). To prepare FE-SEM samples, 5 ml cells was harvested by centrifugation (1 min, 13,000 × *g*), and the pellets were fixed using Karnovsky’s fixation method at 4°C overnight. After cold incubation, the pellets were washed with 0.05 M potassium phosphate buffer three times for 10 min each at 4°C. Dehydration of cells was performed at room temperature using 30%, 50%, 80%, and 100% ethanol serially. The samples were coated with platinum before FE-SEM (FEI, Japan) analysis. Biofilm cells were stained with FilmTracer Sypro Ruby biofilm matrix stain for 30 min at room temperature and observed by CLSM (Carl Zeiss, Germany). Confocal images of stained biofilm were observed under red fluorescent light (excitation wavelength, 450 nm; emission wavelength, 610 nm) to evaluate the height and density of the biofilm.

### Statistical analysis.

The LEfSe algorithm was used to identify significantly different genus or KEGG pathway abundances between the Korean, Chinese, and U.S. and Indian and UAE groups (44). Relative abundance information of genera or KEGG metabolic pathways was imported into LEfSe (version 1.0) on the Web-based Galaxy, and only genera or KEGG metabolic pathways with logarithmic LDA scores of >4.0 (genus) or >3.0 (KEGG) were included. The relative abundances of significantly different taxa between the Korea-China-UAE and Indian and UAE groups were compared through box plot analyses using the package ggplot2, and a Wilcoxon signed-rank test was performed to determine statistical significance. Validation of high, medium, and low biofilm former classification and comparative means of biofilm formation for *Methylobacterium* and non-*Methylobacterium* species were evaluated by Tukey’s test using PROCGLM (SAS). Furthermore, the correlation coefficients and E values between biofilm formation and population size were calculated by Spearman rank correlation.

### Data availability.

The sequenced data for *M. brachiatum* TX0642 were deposited in GenBank (accession numbers CP033231 to CP033234). Pyrosequencing data were also deposited in the Sequence Read Archive (SRA; https://www.ncbi.nlm.nih.gov/sra) under accession numbers SRR7769850 to SRR7769883.
