# Supplementary figures and images for: Dominance of Gas-Eating, Biofilm-Forming Methylobacterium Species in the Evaporator Cores of Automobile Air-Conditioning Systems
Source: mSphere. 2020 Jan 15;5(1):e00761-19. doi: 10.1128/mSphere.00761-19 (PMC6968652; doi:10.1128/mSphere.00761-19)

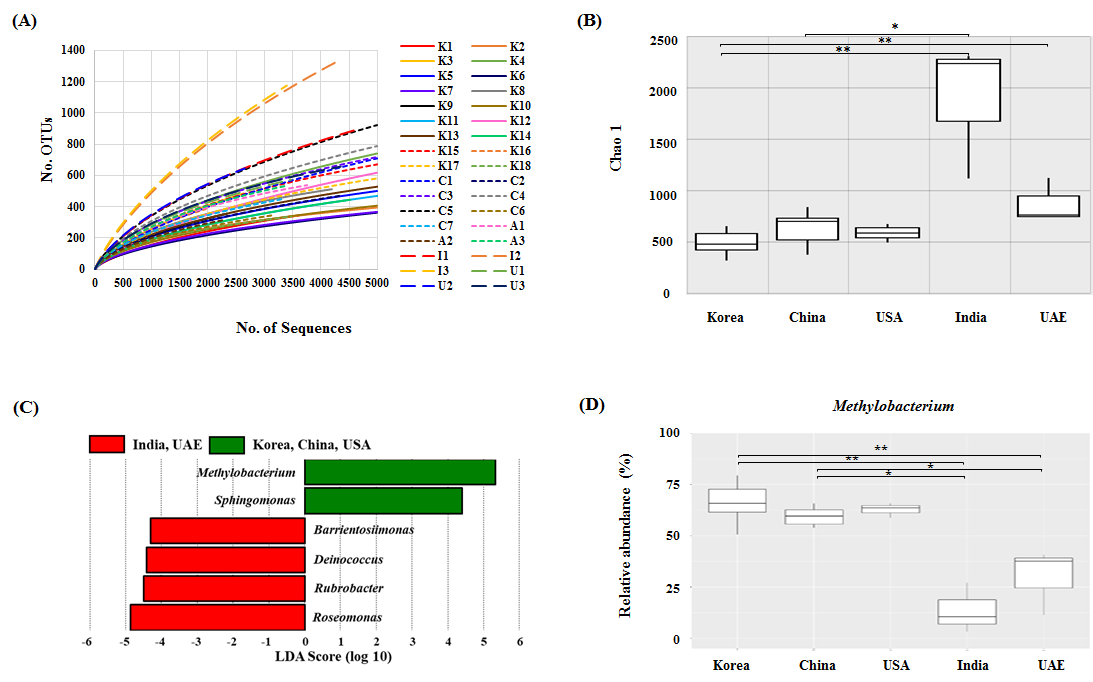

Supplement: FIG S1 [file mSphere.00761-19-sf001.tif]

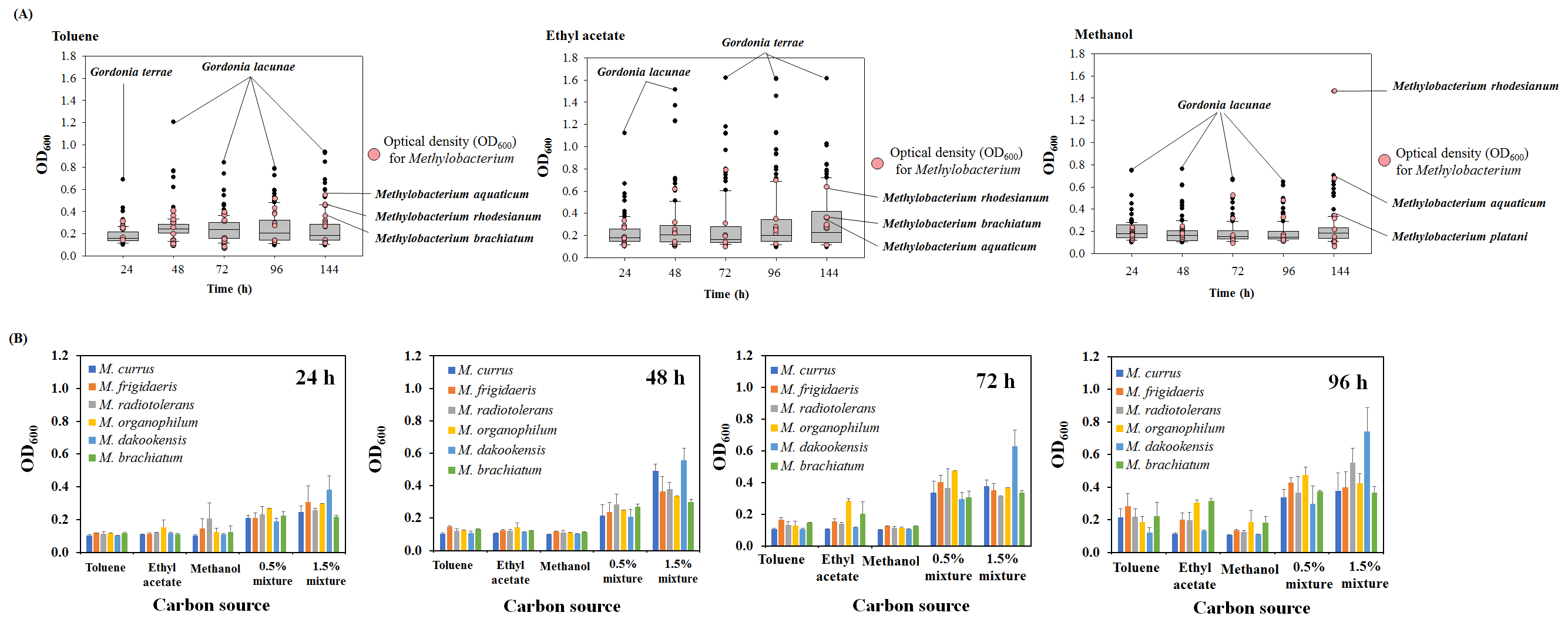

Supplement: FIG S2 [file mSphere.00761-19-sf002.tif]

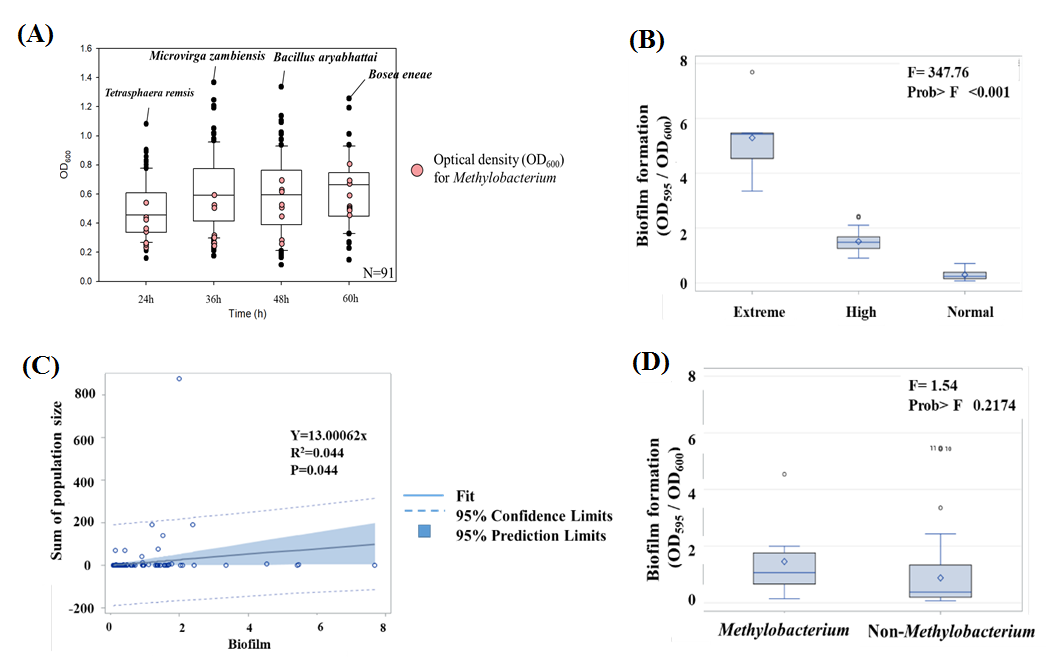

Supplement: FIG S3 [file mSphere.00761-19-sf003.tif]

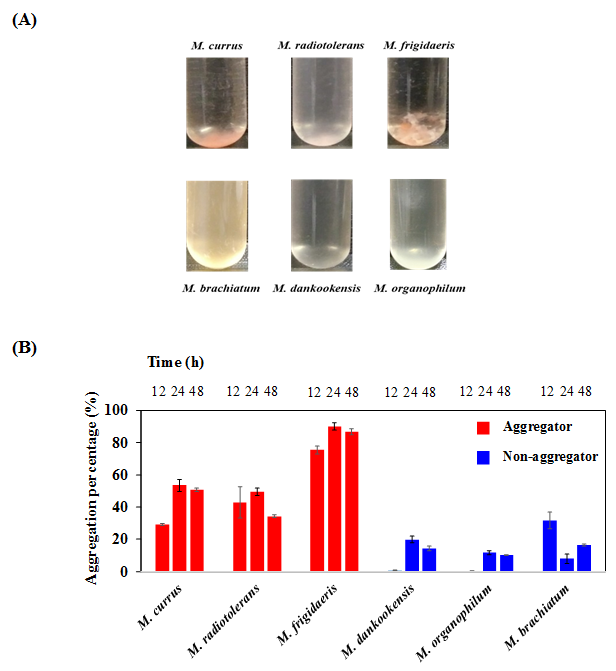

Supplement: FIG S4 [file mSphere.00761-19-sf004.tif]

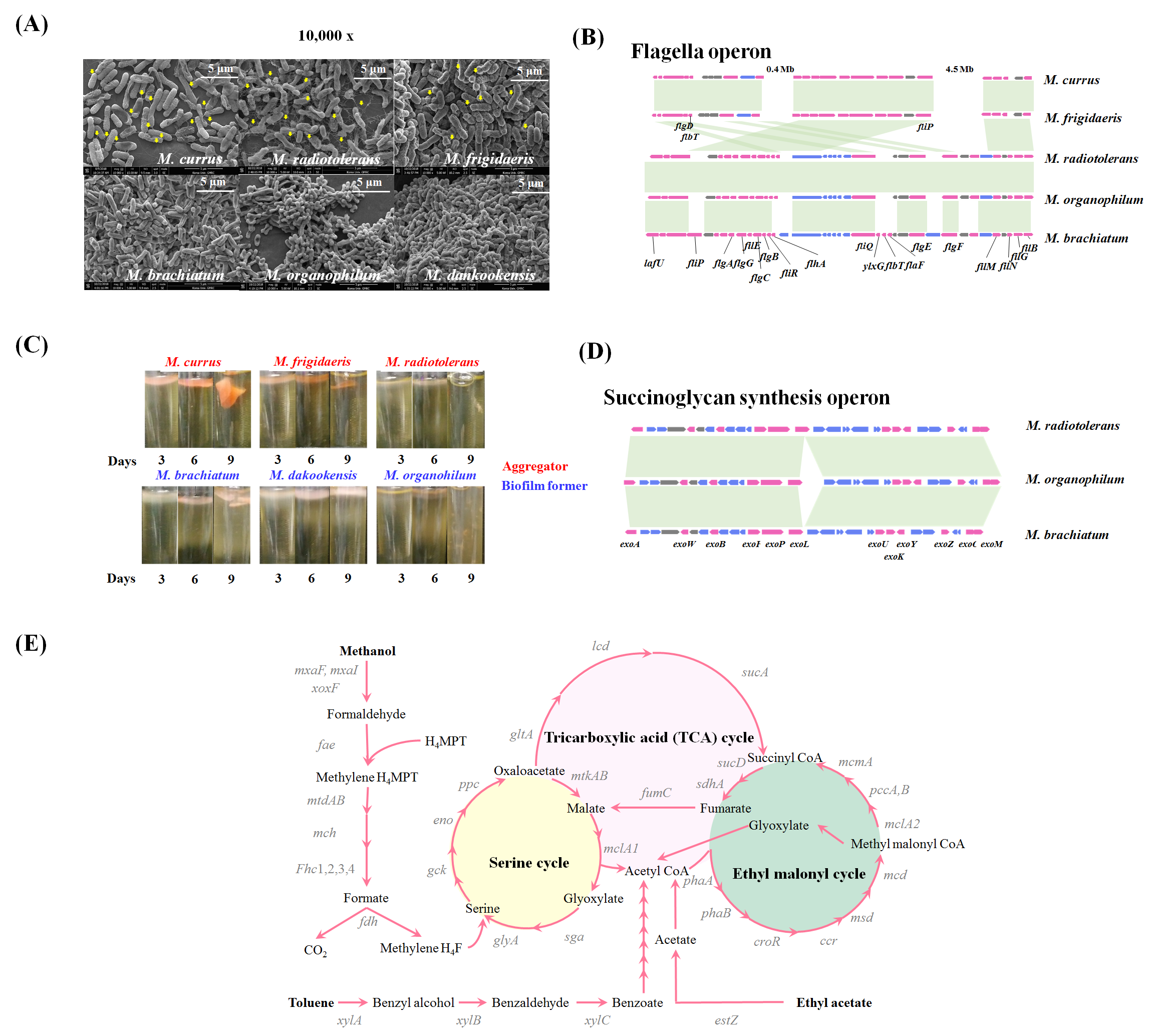

Supplement: FIG S5 [file mSphere.00761-19-sf005.tif]
