# Supplementary material for: Dominance of Gas-Eating, Biofilm-Forming Methylobacterium Species in the Evaporator Cores of Automobile Air-Conditioning Systems
Source: mSphere. 2020 Jan 15;5(1):e00761-19. doi: 10.1128/mSphere.00761-19 (PMC6968652; doi:10.1128/mSphere.00761-19)
Supplement: TABLE S1 [file mSphere.00761-19-st001.pdf]

| Country | Sample name | Auto-dismantling city (sampling city) | Mileage (km) | Car type |
|---|---|---|---|---|
| Korea | K1 | Namyang | 138,000 | Van |
| | K2 | Namyang | 103,998 | Van |
| | K3 | Namyang | 37,000 | Sedan |
| | K4 | Namyang | 69,458 | Coupe |
| | K5 | Namyang | 117,954 | Sedan |
| | K6 | Namyang | 69,400 | Sedan |
| | K7 | Namyang | 61,000 | Hatchback |
| | K8 | Namyang | 84,828 | Sedan |
| | K9 | Namyang | 53,000 | Sedan |
| | K10 | Namyang | 58,420 | Sedan |
| | K11 | Namyang | 39,000 | Sedan |
| | K12 | Namyang | 134,000 | SUV |
| | K13 | Namyang | 24,000 | Sedan |
| | K14 | Namyang | 158,931 | SUV |
| | K15 | Namyang | 110,000 | Sedan |
| | K16 | Ulsan | 62,000 | Hatchback |
| | K17 | Ulsan | 71,550 | SUV |
| | K18 | Ulsan | 18,000 | Sedan |
| China | C1 | Beijing | 33,338 | Sedan |
| | C2 | Beijing | 11,379 | Sedan |
| | C3 | Beijing | 16,287 | Sedan |
| | C4 | Beijing | 23,454 | Sedan |
| | C5 | Beijing | 25,327 | Sedan |
| | C6 | Shanghai | 36,773 | SUV |
| | C7 | Shanghai | 30,877 | Sedan |
| USA | A1 | Irvine, CA | 30,516 | Sedan |
| | A2 | Irvine, CA | 6523 | Sedan |
| | A3 | Irvine, CA | 11,618 | Sedan |
| India | I1 | Delhi | 103,439 | Hatchback |
| | I2 | Delhi | 9598 | Hatchback |
| | I3 | Delhi | 65,111 | Sedan |
| UAE | U1 | Dubai | 209,373 | SUV |
| | U2 | Dubai | 14,863 | Sedan |
| | U3 | Dubai | 40,811 | Sedan |
