# Supplementary material for: Dominance of Gas-Eating, Biofilm-Forming Methylobacterium Species in the Evaporator Cores of Automobile Air-Conditioning Systems
Source: mSphere. 2020 Jan 15;5(1):e00761-19. doi: 10.1128/mSphere.00761-19 (PMC6968652; doi:10.1128/mSphere.00761-19)
Supplement: TABLE S2 [file mSphere.00761-19-st002.pdf]

| Strain | Bacteria | Strain | Bacteria | Strain | Bacteria |
|---|---|---|---|---|---|
| DB1704 | *Afipia broomeae* | TX0407 | *Acidovorax acitrulli* | ID2614S | *Janibacter alkaliphilus* |
| DB2503S | *Bosea eneae* | TX0402S | *Acidovorax avenae* | ID0402 | *Kocuria flava* |
| ID1725 | *Bosea vestrisii* | TX0535 | *Acidovorax oryzae* | MA0202 | *Lapilicoccus jejuensis* |
| TX0646 | *Brandyrhizobium daquingense* | MA1115 | *Acinetobacter johnsonii* | PR1005 | *Leifsonia shinshuensis* |
| DB1509 | *Brandyrhizobium denitrificans* | DB2607S | *Bacillus humi* | MA1109 | *Leifsonia soli* |
| TX0618 | *Brandyrhizobium diazoefficiens* | ID1724 | *Cupriacidus respiraculi* | ID1622 | *Microbacterium aoyamense* |
| TX0647 | *Brandyrhizobium liaoningense* | ID1627 | *Cupriavidus gilardii* | DB0506 | *Microbacterium ginsengisoli* |
| TX0306 | *Brevundimonas vancanneytii* | ID1518 | *Cupriavidus pauculus* | ID2613S | *Microbacterium invictum* |
| TX0632 | *Caulobacter vibrioides* | TX0409 | *Pelomonas puraquae* | ID0631 | *Micrococcus aloeverae* |
| **MA0312** | ***Methylobacterium aerolatum*** | MA1108 | *Pseudomonas nitroreducens* | ID0712 | *Nocardioides ganghwensis* |
| **TX0625** | ***Methylobacterium aquaticum*** | ID1511 | *Lysobacter korlensis* | ID0630 | *Phycicoccus jejuensis* |
| **TX0642** | ***Methylobacterium brachiatum*** | TX0685 | *Fibrella aestuarina* | DB2602S | *Ponticoccus gilvus* |
| **PR1016A** | ***Methylobacterium currus*** | TX0303 | *Chryseobacterium geocarposphaerae* | ID0619 | *Rhodococcus corynebacterioides* |
| **D1702** | ***Methylobacterium dankookensis*** | TX0401S | *Chryseobacterium taichungense* | ID0711 | *Tersicoccus phoenicis* |
| **IER 25-16** | ***Methylobacterium frigidaeris*** | MA1013 | *Flavobacterium oceanosedimentum* | ID0627 | *Tetrasphaera duodecadis* |
| **ID0604** | ***Methylobacterium isbiliense*** | PR1010K | *Mucilaginibacter soli* | ID0724 | *Tetrasphaera remsis* |
| **TX0628** | ***Methylobacterium longum*** | ID0405 | *Bacillus aerophilus* | DB1402 | *Williamsia serinedens* |
| **ID1603** | ***Methylobacterium organophilum*** | PR0007 | *Bacillus amyloliquefaciens subsp. Plantarum* | DB1603 | *Mycobacterium chubuense* |
| **TX0624** | ***Methylobacterium platani*** | MA0003 | *Bacillus aryabhattai* | ID0502 | *Mycobacterium mucogenicum* |
| **TX0706** | ***Methylobacterium radiotolerans*** | PR1102 | *Bacillus licheniformis* | ID2614S | *Janibacter alkaliphilus* |
| **DB0613** | ***Methylobacterium rhodesianum*** | ID0404 | *Bacillus safensis* | | |

| | | | |
|---|---|---|---|
| ID0401 | *Microvirga zambiensis* | ID1406 | *Bacillus simplex* |
| ID0706 | *Novosphingobium pentaromativorans* | PR1104 | *Bacillus subtilis subsp. inaquosorum* |
| TX0530 | *Sphingomonas aquatilis* | MA1102 | *Bacillus subtilis subsp. subtilis* |
| ID1608 | *Sphingomonas desiccabilis* | ID0624 | *Agrococcus lahaulensis* |
| TX0636 | *Sphingomonas dokdonensis* | ID2501S | *Barrientosiimonas humi* |
| TX0724 | *Sphingomonas ginsenosidimutans* | ID2503S | *Brachybacterium conglomeratum* |
| TX0664 | *Sphingomonas ginsenosidivorax* | ID1621 | *Brachybacterium paraconglomeratum* |
| TX0688 | *Sphingomonas glacialis* | DB0623 | *Calidifontibacter indicus* |
| ID1616 | *Sphingomonas kaistensis* | MA1103 | *Curtobacterium flaccumfaciens* |
| TX0714 | *Sphingomonas melonis* | ID1609 | *Dietzia cinnamea* |
| TX0543 | *Sphingomonas mucosissima* | ID1509 | *Georgenia muralis* |
| TX0536 | *Sphingomonas oligophenolica* | ID2629S | *Gordonia lacunae* |
| TX0539 | *Sphingomonas paucimobilis* | ID1610 | *Gordonia terrae* |

- α-Proteobacteria (34)
- β-Proteobacteria (10)
- γ-Proteobacteria (1)
- Bacteriodetes (5)
- Firmicute (8)
- Actinobacteria (33)
