# Supplementary material for: Dominance of Gas-Eating, Biofilm-Forming Methylobacterium Species in the Evaporator Cores of Automobile Air-Conditioning Systems
Source: mSphere. 2020 Jan 15;5(1):e00761-19. doi: 10.1128/mSphere.00761-19 (PMC6968652; doi:10.1128/mSphere.00761-19)
Supplement: TABLE S3 [file mSphere.00761-19-st003.pdf]

Class	Sample no.	Bacteria	Biofilm ability value (OD_{600}/OD_{595})
Extreme	46	*Sphingomonas paucimobilis*	7.7
	94	*Lysobacter korlensis*	5.4
	21	*Sphingomonas ginsenosidimutans*	5.5
	97	*Methylobacterium organophilum*	4.5
	107	*Micrococcus aloeverae*	3.4
High	84	*Brachybacterium paraconglomeratum*	2.4
	18	*Sphingomonas melonis*	2.4
	125	*Methylobacterium tardum*	2.3
	82	*Bosea vestrisii*	2.1
	3	*Brandyrhizobium liaoningense*	2.0
	13	*Methylobacterium longum*	2.0
	45	*Sphingomonas oligophenolica*	1.8
	117	*Tersicoccus phoenicis*	1.7
	104	*Nocardioides ganghwensis*	1.7
	78	*Bacillus safensis*	1.7
	81	*Bosea eneae*	1.6
	98	*Microbacterium aoyamense*	1.6
	93	*Kocuria flava*	1.6
	77	*Bacillus aerophilus*	1.6
	15	*Methylobacterium platani*	1.5
	9	*Sphingomonas mucosissima*	1.4
	23	*Bacillus humi*	1.4
	118	*Tetrasphaera duodecadis*	1.4
	35	*Williamsia serinedens*	1.4
	38	*Brevundimonas vancanneytii*	1.4
	6	*Caulobacter vibrioides*	1.3
	88	*Cupriacidus respiraculi*	1.3
	12	*Methylobacterium aquaticum*	1.2
	122	*Methylobacterium brachiatum*	1.1
	44	*Sphingomonas aquatilis*	1.1
	87	*Cupriavidus pauculus*	1.0
	79	*Bacillus simplex*	1.0
	43	*Sphingomonas ginsenosidivorax*	0.9
	96	*Methylobacterium isbiliense*	0.9
	123	*Methylobacterium dankookensis*	0.9
	54	*Methylobacterium aerolatum*	0.9
