# Supplementary material for: Dominance of Gas-Eating, Biofilm-Forming Methylobacterium Species in the Evaporator Cores of Automobile Air-Conditioning Systems
Source: mSphere. 2020 Jan 15;5(1):e00761-19. doi: 10.1128/mSphere.00761-19 (PMC6968652; doi:10.1128/mSphere.00761-19)
Supplement: TABLE S4 [file mSphere.00761-19-st004.pdf]

(Unit: %)

	**Fucose**	**Galactose**	**Glucose**	**Mannose**	**Rhamnose**
M. currus	0.6	0.0	25.3	73.2	0.9
M. brachiatum	0.6	15.7	20.4	63.2	0.0
