# Supplementary material for: Dominance of Gas-Eating, Biofilm-Forming Methylobacterium Species in the Evaporator Cores of Automobile Air-Conditioning Systems
Source: mSphere. 2020 Jan 15;5(1):e00761-19. doi: 10.1128/mSphere.00761-19 (PMC6968652; doi:10.1128/mSphere.00761-19)
Supplement: TABLE S5 [file mSphere.00761-19-st005.pdf]

Table S5. List of adaptor and barcode sequences in PCR primer sets used in this study.

Name	Sequence (5′-3′)	Reference
Adaptor sequence		
A adaptor	CCATCTCATCCCTGCGTGTCTCCGACTCAG	Roesch et al., 2007[1]
B adaptor	CCTATCCCCTGTGTGCCTTGGCAGTCTCAG	Roesch et al., 2007
Universal primers		
9F	GAGTTTGATCMTGGCTCAG	Nam et al, 2011[2]
541R	WTTACCGCGGCTGCTGG	Nam et al, 2011
Sample	Barcode (5'-3')	
K1	CTGTCTACG	
K2	TGACTCTG	
K3	CAGCTAGT	
K4	AGCTGTG	
K5	ACACTGTG	
K6	AGTCACTAG	
K7	CAGCATG	
K8	CTGTCTACG	
K9	CGTGTACTG	
K10	ACGTGCAGCG	
K11	AGCTATCGCG	
K12	CTAGCTG	
K13	ATCGTGTG	
K14	ATCACGTGCG	
K15	ATAGCTCTCG	
K16	ATGCTGAG	
K17	CTACACAG	
K18	ACACTGTG	
C1	ATCGTGTG	
C2	TACAGCAG	
C3	CTGTCTACG	
C4	ATGCTGAG	
C5	CGTGTACTG	
C6	TACAGCAG	
C7	AGACAGTACAG	
A1	ATCGTGTG	
A2	TACAGCAG	
A3	CTGTCTACG	
I1	ACACACTAG	
I2	TACGTAGACAG	
I3	CGAGATAGATG	
U1	ACTCGATG	
2	ATCGACAG	
U3	ATGTACACG	

1. Roesch LF, Fulthorpe RR, Riva A, Casella G, Hadwin AK, Kent AD, Daroub SH, Camargo FA, Farmerie WG, Triplett EW. 2007. Pyrosequencing enumerates and contrasts soil microbial diversity. ISME J 1:283–290.
2. Nam YD, Jung MJ, Roh SW, Kim MS, Bae JW. 2011. Comparative analysis of Korean human gut microbiota by barcoded pyrosequencing. PLoS One 6:e22109.
